# NGSmirPlant: comprehensive characterization of the small RNA transcriptomes of plants

**DOI:** 10.1007/s13238-015-0159-z

**Published:** 2015-05-09

**Authors:** Jie Bai, Chen Dan, Yi Zhang, Guoping Zhao, Xiaoming Ding

**Affiliations:** Department of Microbiology and Microbial Engineering, School of Life Sciences, Fudan University, Shanghai, 200433 China; Key Laboratory of Synthetic Biology, Institute of Plant Physiology and Ecology, Shanghai Institutes for Biological Sciences, Chinese Academy of Sciences, Shanghai, 200032 China; Shanghai-MOST Key Laboratory of Health and Disease Genomics, Chinese National Human Genome Center at Shanghai, Shanghai, 201203 China

MicroRNAs (miRNAs) are short, endogenously expressed RNAs (~21–22 nt) processed from stem-loop regions of longer RNA precursors by a Dicer-like enzyme (Jones-Rhoades et al., 2006). In plants, microRNAs play an essential role in regulating many fundamental molecular interactions, including plant growth, development and response to environmental stress (Rogers and Chen, 2013). In comparison with the roles of microRNAs in animals, biogenesis and function of microRNAs in plants appears to be drastically different (Llave et al., 2002; Zhang et al., 2007). For example, prevalent in animal, microRNAs negatively regulate gene expression by attenuating translation. In contrast, direct microRNA-guided mRNA cleavage occurs in plant kingdom. In addition, for microRNA induced regulation in animal, translation is inhibited by microRNA with imperfect base-pairing to target mRNA. In plant, however, microRNAs inhibit the translation of target mRNA through extensively complementarity to its targets (Wu, 2013).

MicroRNAs can be identified by the approaches of hybridization-based experimental and bioinformatics prediction followed by experimental validation (Jones-Rhoades et al., 2006). With the limitations of experimental methods, microRNAs of low expression level are very difficult to be identified, causing certain microRNA to be missed. Bioinformatics approaches are often associated with a relatively high false negative and false positive rate. Thus, so far, due to limit of these methods, only a small number of plant microRNAs have been identified. Nowadays, with the advent of next-generation sequencing technologies, such as Roche 454, Illumina Solexa and ABI SOLiD, small RNA transcriptome research has been revolutionized. Compared to previously reported methods, it has distinct advantages of in-depth analysis of small RNA populations and the ability to identify novel microRNAs (Creighton et al., 2009; Fahlgren et al., 2009; Tam et al., 2014). However, deep sequencing technologies have generated hundreds of thousands of reads with large amounts of data, which give us substantial bioinformatics challenges to mine the hidden biological significance.

Currently, several useful tools have been developed to allow users to investigate the microRNAs from deep sequencing data, such as miRanalyzer (http://web.bioinformatics.cicbiogune.es/microRNA/), miRExpress (http://miRExpress.mbc.nctu.edu.tw), mirTools (http://centre.bioinformatics.zj.cn/mirtools/), miRNAkey (http://ibis.tau.ac.il/miRNAkey), DSAP (http://dsap.cgu.edu.tw), mirDeep* (http://www.australianprostatecentre.org/research/software/mirdeep-star), miREval 2.0 (http://mimirna.centenary.org.au/mireval/). However, except the UEA small RNA tool (http://srna-tools.cmp.uea.ac.uk), few tools are available for identifying plant microRNAs due to important differences of existed reference resources, mode of action and search criteria. Herein, a central motivation for this study is to specifically develop a comprehensive tool in an effort to easily investigate the large amount of NGS data for plant microRNAs. We describe a new web-based resource NGSmirPlant for the identification, characterization and differential expression analysis of microRNAs, as well as detection of novel microRNAs. Compared with the UEA small RNA tool, NGSmirPlant provides detailed annotation information for the comparison between mature microRNAs and users-input microRNAs, such as absolute counts, relative counts and sequences. In addition, NGSmirPlant implements an evolutional conservation analysis for all known microRNA families included in the result among a set of plant species. The interface of NGSmirPlant is easy to use and will be extremely helpful to plant molecular biologists with amateur bioinformatics skills to investigate the plant small RNA transcriptome.

Different small RNA has different length, which is due to distinct mechanisms of its biogenesis process. Through a read filter process, NGSmirPlant will obtain the unique length distribution of small RNA transcriptome obtained from deep sequencing, which can be used to distinguish the different classes of small RNAs and estimate the performance of sequencing library constructed. In this study, NGSmirPlant provides two kinds of length distribution (Fig. 1): one is based on unique tag and the other is based on tag abundance. The unique tag distribution is only used for estimation of the diversity of unique tag obtained from the library. The tag abundance distribution can be used to estimate the performance of sequencing library. For example,microRNAs are the most abundant class of small RNAs in plant, which will has a peak at the 21 or 22 nucleotides lengths from Dicer digestion products (Jones-Rhoades et al., 2006).

**Figure 1:**
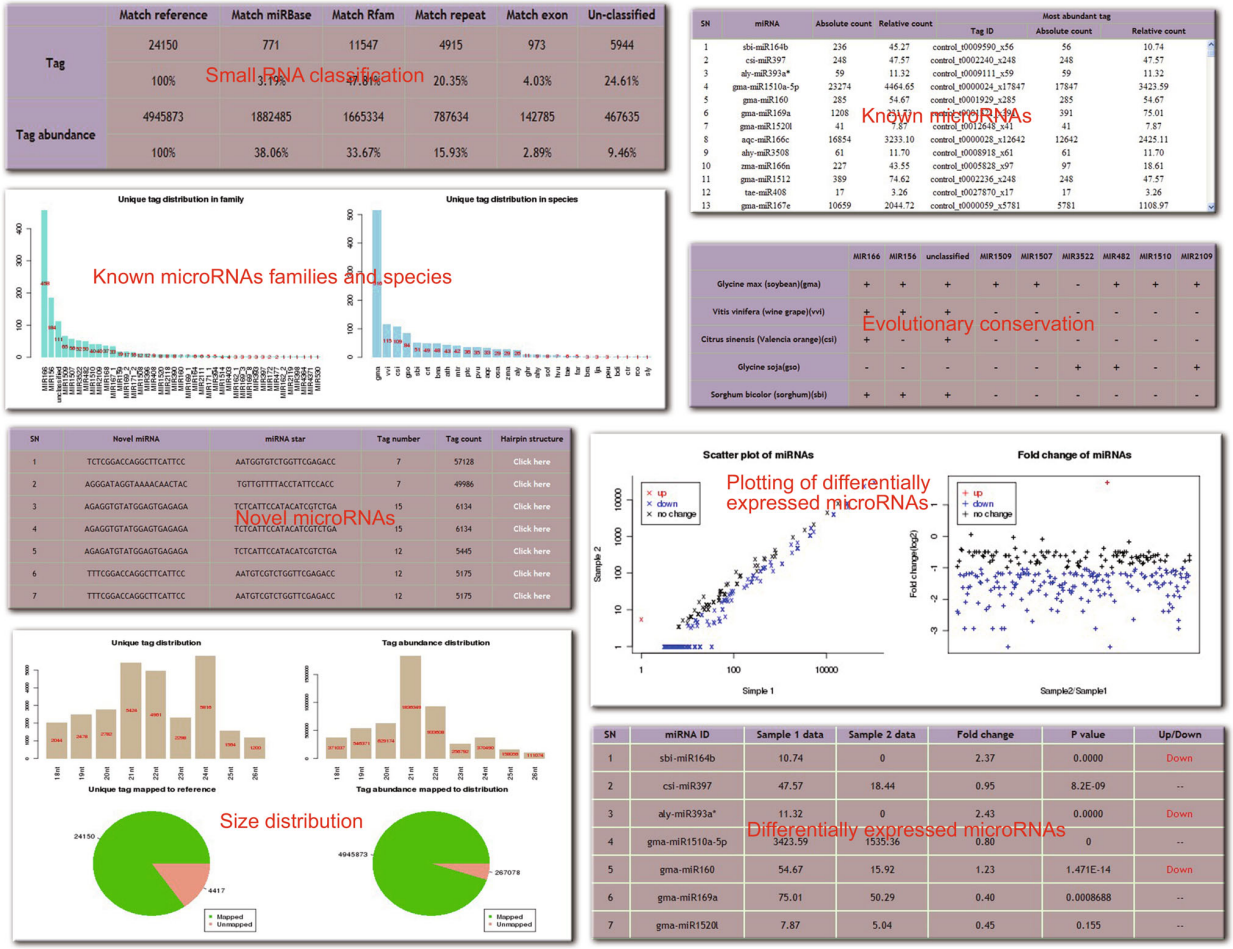
**The typical output of NGSmirPlant web server**. It includes: 1) the size distribution of small RNA transcriptome; 2) the classification of small RNAs known microRNAs, repeat-associated RNA, the ncRNAs reads annotated by Rfam (rRNA, tRNA, snRNAs and snoRNA et al.) and mRNA degradation products; 3) the known and novel microRNAs identification; 4) evolutionary conservation of individual microRNA in various plant species; and 5) differentially expressed microRNAs between samples

Recent studies have revealed that small RNA transcriptome is far more complex than previously thought, which might play important roles in the physiology of the cell (Creighton et al., 2009; Fahlgren et al., 2009; Tam et al., 2014). Deep sequencing has offered an exciting opportunity to determine the complexity of small RNA transcriptome in the mean of single-base resolution, genome-wide scale and cost-effectiveness. Initially, NGSmirPlant performed the mapping of sequence tags mapping to the reference genome or EST using SOAP 2.0 (http://miRExpress.mbc.nctu.edu.tw). It is found that, besides high abundance of microRNA, non-coding small RNAs (snRNAs, snoRNAs and tRNAs et al. obtained from the Rfam database) will also occupy many sequence tags even for the specific designed microRNA sequencing library (http://122.228.158.106/NGSmirPlant/download1.php?id=1414376897). In addition, a small proportion of sequencing tags could be mapped to exon sequences, which are likely to be RNA degradation products. Therefore, the aligned reads are classified into known microRNAs, repeat-associated RNA, the ncRNAs reads annotated by Rfam (rRNA, tRNA, snRNAs and snoRNA et al.) and mRNA degradation products (Fig. 1). If a sequence tag has multiple hits, we only assign it a single category based on the following priority: ncRNA from Rfam, known plant microRNA, repeat-associated RNA and mRNA degradation product. Through such a way, NGSmirPlant will identify the expression level of each kind of small RNA by calculating the number of total tags it belongs to.

One of the most important advantages for deep sequencing of small RNA transcriptome is that it can be used for the discovery of novel microRNAs (Creighton et al., 2009). In NGSmirPlant, to identify candidate novel microRNAs, we first filter out sequence tags that have been classified into annotated categories, such as non-coding RNA and known microRNAs. With the implement of the miRDeep program (Friedlander et al., 2008), NGSmirPlant provides candidate novel microRNAs with the RNA secondary structure, minimal free energy, tag counts of mature/mature* sequence et al. (Fig. 1). Users can use other method, such as hybridization-based experimental method, to validate the reliability of novel microRNA identified.

Since many microRNA families are evolutionarily conserved across many lineages in the plant kingdom and the fact that lots of microRNAs in different plant species shared the same mature sequence in miRBase 19.0 (http://microrna.sanger.ac.uk/), microRNA genes in one species may have orthologs or homologs existed in other species. Given that, we enlarged the annotation scale to an expansion of multiple species of known and novel microRNAs. For the identified microRNAs, the measurement of its evolutionary conservation was performed by similarity search against the currently known microRNAs in miRBase 19.0 in other plant species (Fig. 1). If a read matched one or more known microRNAs with >95% identity, it was regarded as homologous in evolution. Through the evolutionary conservation profile, the lineage-specific and species-specific microRNAs can be identified and visualized easily.

NGSmirPlant employs two different strategies to investigate the microRNAs expression profile and detect the differentially expressed microRNAs between different libraries (Fig. 1). One is based on the total tag count (#specific microRNA tags/#total microRNA sequence tags) and the other is based on the most abundant tag count (#the most abundant tag of specific microRNA/#total microRNA sequence tags). Each identified microRNA sequence numbers were normalized to the total number of microRNA read counts followed by multiplying one million to obtain reads per million (RPM) value. The statistical significance (*P*-value) is inferred based on the Bayesian method (Audic and Claverie, 1997). Flexibly, the threshold for fold change as differentially expressed microRNA is user-definable. NGSmirPlant provides differentially expressed microRNAs with microRNA ID, relative sequenced count, fold change, increase/decrease and *P* value.

The web server of NGSmirPlant is well organized in a user-friendly access manner. It runs with the input of a single sample or multiple samples. For “single” mode, NGSmirPlant will analyze and annotate small RNAs embed in the sample and give a list of novel microRNA details. For “multiple” mode, the differentially expressed microRNAs between the pair samples will be in-depth investigated additionally.

The web server input of NGSmirPlant is pre-processed FASTA format where all the unique sequence tags aggregated with a number behind the name to represent the expression level and cleaned into a non-redundant FASTA file to reduce the input size. The users can download the Perl script in the filter column of web server to a local client for generating the proper input format of NGSmirPlant. The size of input file should be limited to 10 MB, which can be in FASTA format or compressed in zip or gz format of FASTA file (Fig. 2). A demo for the web server is provided. Users can download the example input file for “single” or “multiple” mode and run the tools with default parameters. In general, a typical “single” run takes about 20 min and a typical “multiple” run takes about 40 min.

**Figure 2:**
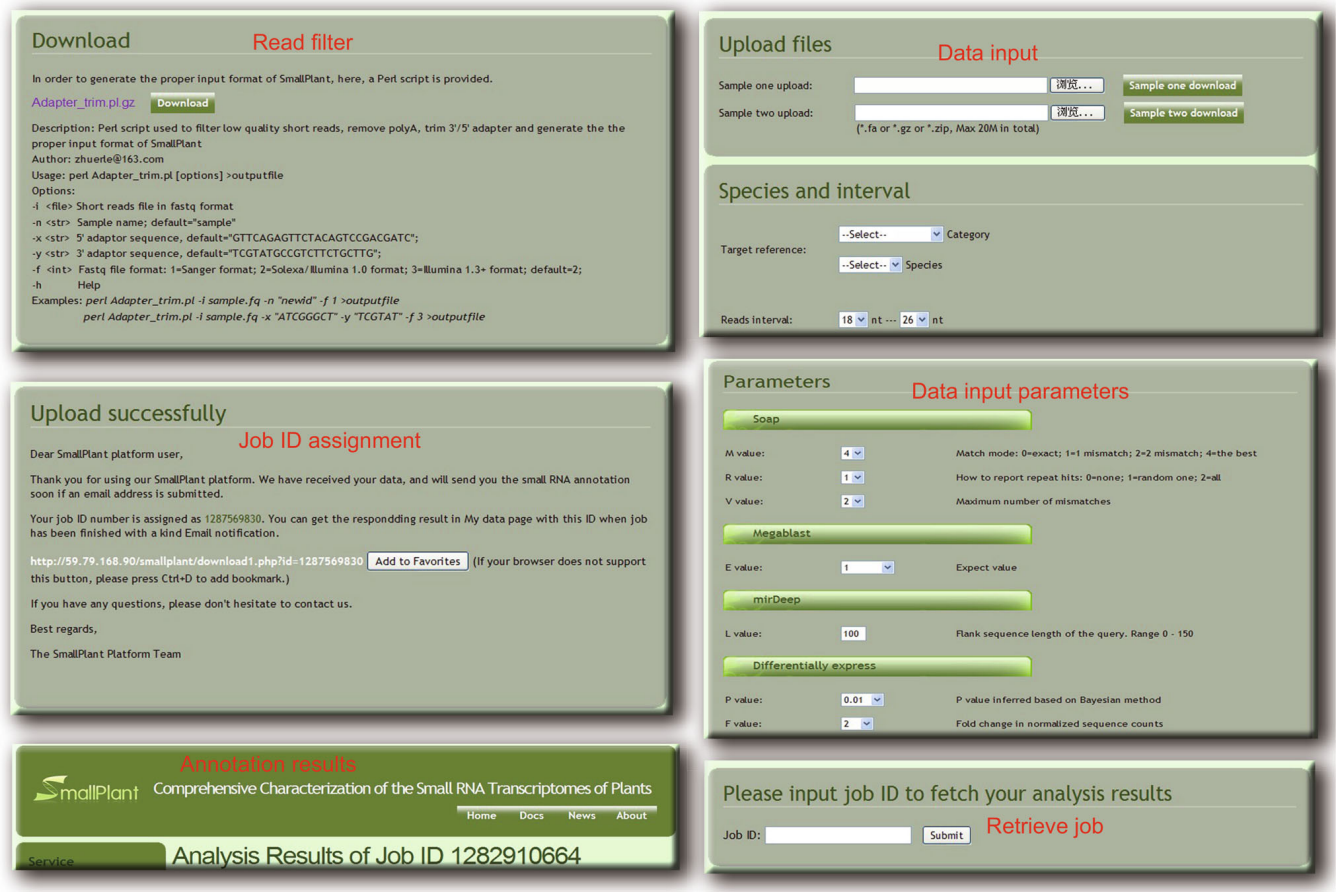
**The typical input of NGSmirPlant showing the reads filter requirement, file input, job ID assignment and job retirement**

NGSmirPlant provide a number of important parameters in both of “single” mode and “multiple” mode, so users can set these parameters by themselves (Fig. 2). Users can set the reads length interval (default is 18~30 nt) thus the tag sequences longer or shorter than the threshold will be discarded. During the alignment mapping process, a set of parameters are available for users to define. With the “R value” parameter, users can freely define the strategy for reporting repeat hits. It should be noticed that the “M value” parameter denotes the maximum number of allowed mismatches for the seed part of a read (at most two mismatches) while the “V value” parameter means the total allowed mismatches in one read. When predicting novel microRNAs, the expected value and the flanking sequence length of the query can be defined by users. For “multiple” mode, two parameters for differentially expression are provided. By setting the threshold of *P* value inferred based on Bayesian method and fold change in normalized sequence counts, the specific microRNA satisfied both the *P*-value and the fold change will be deemed to be significantly differentially expressed in the two samples (Fig. 3).

**Figure 3:**
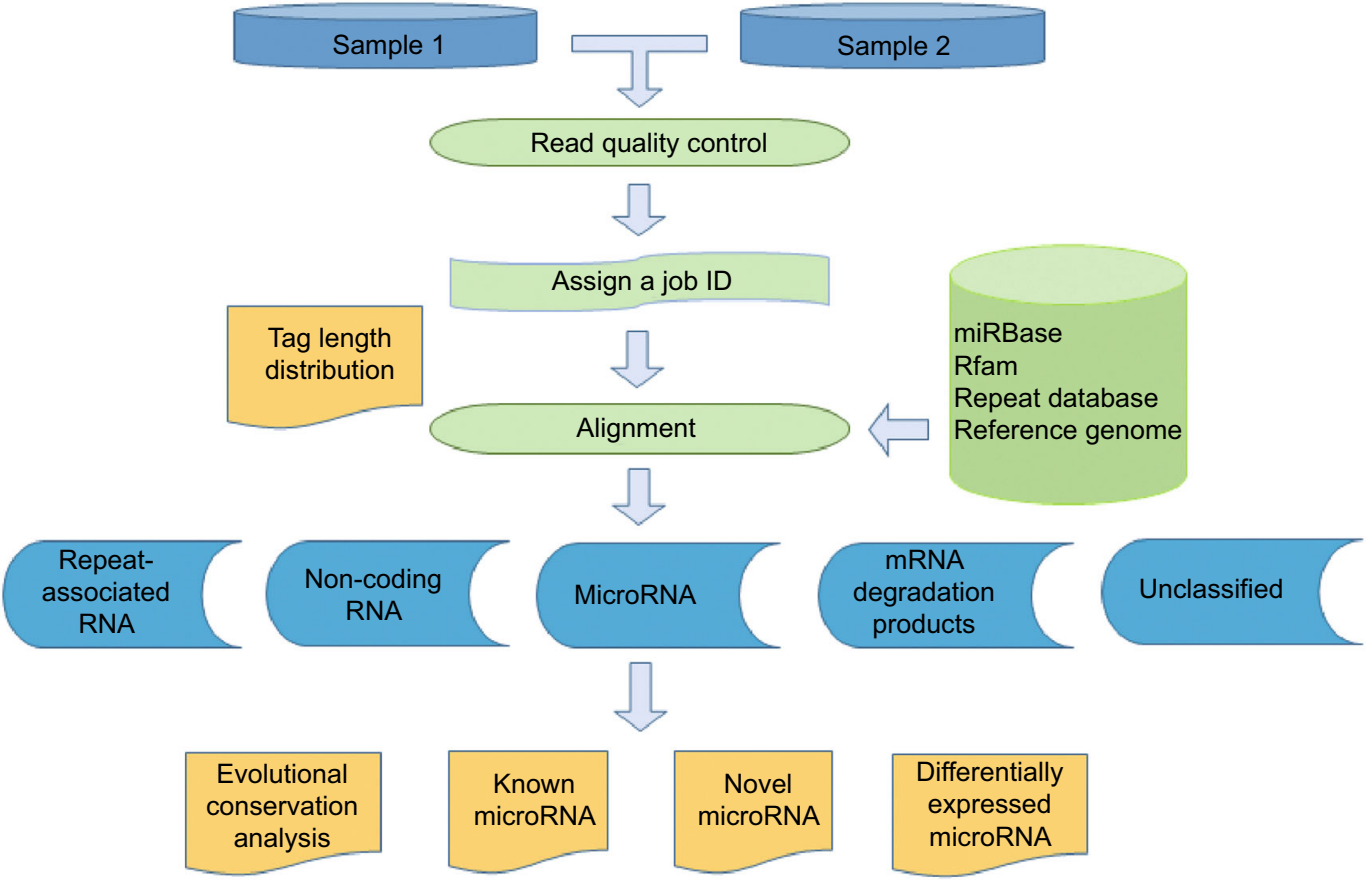
The workflow of NGSmirPlant. In brief, NGSmirPlant firstly performs the read quality control and aligns the clean reads to a number of reference databases, such as genomic sequences or ESTs, miRBase, Rfam and repeat database produced by RepeatMasker. The small RNA annotation categories will be then identified, include microRNAs, repeat-associated RNA, non-coding RNA and mRNA degradation products. Meanwhile, NGSmirPlant will provide detailed annotation of known and novel microRNAs, differentially expressed microRNA between samples and its evolutional conservation

Once a job is submitted successfully, NGSmirPlant will be assigned with a unique job ID and users need to wait for the result by e-mail provided. Then, users will receive a notification e-mail with a URL link automatically. Users can use this unique ID to visit the annotation results or retrieve previous job, which will be kept one month in NGSmirPlant. The job ID submitted older than one month will be deleted. Subsequently, NGSmirPlant will show: 1) basic summary information of library, such the length distribution of unique sequence size, which can be used to indicate the most abundant of small RNA category in the library; 2) alignment information indicating the tag abundance matched to reference genome, miRBase, Rfam, repeat, exon and unannotation respectively; 3) detailed annotation information of the known microRNA identified, such species and families distribution, the absolute (total number of sequence tags mapped to a specific microRNA) and relative (normalization of matched read counts to the total number of microRNA reads and then multiplied by a million) expression level and evolutionary conservation of individual microRNA in various plant species; 4) detailed annotation information of other non-coding RNA identified, such as rRNA, tRNA, snRNAs and snoRNA; 5) detailed annotation information of novel microRNA identified, such as novel microRNA sequence, microRNA star and hairpin structure; 6) detailed information of microRNA different expression. All these results can be downloaded from NGSmirPlant to allow users to perform easy visualization or further analysis in the local computer.

The small RNAs, especially the microRNAs, play important roles in different biological processes in plant, such as plant growth, development and response to environmental stress. However, currently, only a small number of plant microRNAs have been detected due to the limitation of hybridization-based experimental and bioinformatics prediction methods. However, the recently developed deep sequencing technologies have been proved to have distinct advantages of unbiased and in-depth analysis of the complexity of small RNA transcriptome and its powerful ability to identify known and novel microRNAs. However, next-generating sequencing technologies have produced hundreds of thousands of short reads with large amounts of data, which give us substantial bioinformatics challenges to discover the hidden biological significance. Therefore, in this study, NGSmirPlant (http://122.228.158.106/NGSmirPlant/) is specifically designed towards a web-based server to fulfill the urgent need for bioinformatics analysis of plant small RNA transcriptome by deep sequencing. In general, NGSmirPlant can be used to facilitate a genome-wide analysis of microRNAs profiling and discovery, exploring evolutionary conservation of individual microRNA in various plant species.

In comparison with other microRNA profiling tools, such as mirTools (http://centre.bioinformatics.zj.cn/mirtools/) and CPSS (http://mcg.ustc.edu.cn/db/cpss/index.html) which are either specialized for animal small RNAs analysis or incomplete analysis of plant small RNAs, NGSmirPlant is specialized for comprehensive characterization of the small RNA transcriptome of plants. Currently, it covers a broad range of 85 plant organisms from the KEGG database (http://www.genomejp/kegg/catalog/org_list.html, includes completely finished genomic sequences and the available ESTs) to allow users to effectively identify known and novel microRNAs. It is believed that the comprehensive integration of plant species could meet the need of most researchers who involve in plant small RNA analysis from deep sequencing. Moreover, NGSmirPlant performs evolutionary conservation analysis for each microRNA family while mirTools and CPSS do not. In this part, those relatively conserved microRNA families among different species are listed with a symbol “+”. In addition, the detailed annotation of known microRNAs is performed for the comparison between mature microRNAs and users-input microRNAs, such as absolute counts, relative counts and sequences. Compared with CPSS, NGSmirPlant exhibits more readable graphs, such as the detailed distribution of unique tags in families and species, which provides a more concise result for users to visualize the results. As with other microRNA profiling tools, such as the UEA small RNA tool, NGSmirPlant also perform an analysis of differentially expressed microRNAs between samples. Meanwhile, it is very useful that NGSmirPlant draws scatter plots for total tag count and the most abundant tag, which makes the expression differentiation extremely clear.

In the future version, we will continually integrate more number of finished plant genomes or ESTs data as they become increasingly available. In addition, the pre-processing script provided in the current version only supports with Illumina sequencing data and as such would require users to develop their own pre-processing steps in order to use 454 or SOLiD data. In the future, a more general and flexible pre-processing script will be made available that would accept the raw data obtained from the three common used high-throughput sequencing platforms and generate the required format for NGSmirPlant input. To further improve on the power of NGSmirPlant, future development will implement the microRNA target prediction tools to predict the target genes of identified microRNAs in plant. Moreover, in addition to the microRNA target prediction tools, NGSmirPlant will plan to perform detailed functional annotation of microRNA targeted genes, including Gene Ontology, KEGG pathway and protein-protein interaction. Through which, NGSmirPlant will provide the opportunities to elucidate the complex microRNAs mediated regulatory networks. NGSmirPlant welcomes any questions, comments and suggestions, which will be a useful feedback for future updating. In conclusion, we believe that NGSmirPlant will be a straight-forward valuable resource that are opening fascinating opportunities for in-depth investigation of small RNA transcriptome generated from next-generation sequencing technologies.

## Electronic supplementary material

Supplementary material 1 (PDF 140 kb)
