# Supplementary material for: NGSmirPlant: comprehensive characterization of the small RNA transcriptomes of plants
Source: Protein Cell. 2015 May 9;6(6):397–402. doi: 10.1007/s13238-015-0159-z (PMC4444805; doi:10.1007/s13238-015-0159-z)
Supplement: Supplementary file 1 — Supplementary material 1 (PDF 140 kb) [file 13238_2015_159_MOESM1_ESM.pdf]

Supplementary materials

Materials and methods

The workflow of NGSmirPlant is shown in Figure 3. For the deep sequencing reads generated by Illumina sequencer, firstly, NGSmirPlant provides an easy-to-use script in the filter column of the web server for filtering out low-quality reads, 3'/5' adaptor sequences and poly A sequences. Low quality reads are filtered using Trim Galore (http://www.bioinformatics.babraham.ac.uk/projects/trim_galore/). The 3'/5' adapters and poly A sequences are trimmed using the Cutadapt program (http://code.google.com/p/cutadapt/) implemented in the Trim Galore program. Only reads with the sequencing quality is greater than 20 and read length is greater than 16bp are retained. The script runs in a local computer both in Linux and Windows operating system, which will significantly reduce the input size to avoid of uploading the raw fastq data. Then, those reads passing through the filtering criteria are trimmed into clean full-length reads. Each unique sequence read (the sequence of a particular type with non-redundancy) is counted as sequence tag with its counting number representing the relative abundance. For example, for the unique tag named sample-240x180, sample-240 represents a unique ID and 180 is the read count representing the expression level. Subsequently, a non-redundant formatted FASTA file will be obtained.

To identity known plant microRNA from the deep sequencing data, sequence tags are firstly aligned to the known plant microRNAs in miRBase 19.0 (http://microrna.sanger.ac.uk/) using SOAP 2.0 (http://miRExpress.mbc.nctu.edu.tw)

with at most two mismatches (Figure 3). Poor matched read tags which do not satisfy mapping parameters selected from the genome or ESTs are ignored, while eligible matches are retained as candidates for further annotations. The annotation categories include known microRNAs, repeat-associated RNA, the ncRNAs reads annotated by Rfam (rRNA, tRNA, snRNAs and snoRNA et al.) and mRNA degradation products. All unique sequence tags are aligned against miRBase, Rfam (http://rfam.xfam.org/), repeat database produced by RepeatMasker (Tarailo-Graovac and Chen, 2009) and the coding genes of the reference genome downloaded from phytome (Stefanie, et al., 2006) respectively.

To identify candidate novel microRNAs, we filter out sequence tags that have been classified into annotated categories, such as non-coding RNA and known microRNAs. Then, the remaining tags are re-aligned with its genome or ESTs using the SOAP 2.0 program (http://miRExpress.mbc.nctu.edu.tw) with at most two mismatches. User-definable length (100 at default) of genomic sequence flanking each side of unclassified sequences is extracted from the genome or ESTs. The small RNA secondary structure of putative precursor is predicted using the RNAfold program (Hofacker, 2003). Finally, novel microRNAs are predicted with the implement of the miRDeep program (Friedlander et al., 2008), among which the occurrence of both a microRNA and a microRNA* in the deep sequencing data is the basis to predict novel microRNAs.

NGSmirPlant is established based on a number of open source software, including Apache, PHP and Perl, etc. In brief, the web server is based on Apache

HTTP server and its pages are produced through a combination of PHP language and Perl CGI scripts. Meanwhile, the BioPerl modules are applied to manipulate data and convert different data formats. The plots are generated by R plot packages. All the procedures above are executed on the Linux operating system. NGSmirPlant has developed a queuing system to track the user-submitted jobs. It executes two jobs in parallel and the remainder will be put into the queue to wait the previous job to be finished. The web client of NGSmirPlant is designed to be independent of operating systems and has been successfully tested under different versions of Linux, Microsoft Windows, and MacOS with Microsoft Internet Explorer 6.0, Firefox 2, Google Chrome 22.0, and Safari 5.0.
